# NIR self-powered photodetection and gate tunable rectification behavior in 2D GeSe/MoSe_2_ heterojunction diode

**DOI:** 10.1038/s41598-021-83187-z

**Published:** 2021-02-11

**Authors:** Muhammad Hussain, Syed Hassan Abbas Jaffery, Asif Ali, Cong Dinh Nguyen, Sikandar Aftab, Muhammad Riaz, Sohail Abbas, Sajjad Hussain, Yongho Seo, Jongwan Jung

**Affiliations:** 1 Department of Nanotechnology and Advanced Materials Engineering, and HMC, Sejong University, Seoul, 05006 South Korea; 2 Department of Engineering, Simon Faster University, Burnaby, Canada; 3 Faculty of Engineering and Applied Sciences, Ripah International University, Islamabad, Pakistan

**Keywords:** Engineering, Materials science, Nanoscience and technology, Optics and photonics

## Abstract

Two-dimensional (2D) heterostructure with atomically sharp interface holds promise for future electronics and optoelectronics because of their multi-functionalities. Here we demonstrate gate-tunable rectifying behavior and self-powered photovoltaic characteristics of novel p-GeSe/n-MoSe_2_ van der waal heterojunction (vdW HJ). A substantial increase in rectification behavior was observed when the devices were subjected to gate bias. The highest rectification of ~ 1 × 10^4^ was obtained at V_g_ = − 40 V. Remarkable rectification behavior of the p-n diode is solely attributed to the sharp interface between metal and GeSe/MoSe_2_. The device exhibits a high photoresponse towards NIR (850 nm). A high photoresponsivity of 465 mAW^−1^, an excellent EQE of 670%, a fast rise time of 180 ms, and a decay time of 360 ms were obtained. Furthermore, the diode exhibits detectivity (D) of 7.3 × 10^9^ Jones, the normalized photocurrent to the dark current ratio (NPDR) of 1.9 × 10^10^ W^−1^, and the noise equivalent power (NEP) of 1.22 × 10^–13^ WHz^−1/2^. The strong light-matter interaction stipulates that the GeSe/MoSe_2_ diode may open new realms in multi-functional electronics and optoelectronics applications.

## Introduction

Beyond the great success of graphene and their derivatives, the analogs of the 2D materials, such as the transition metal dichalcogenide (TMD) and the transition metal carbide (TMC) nanostructures have strikingly increased interest in science. Compared to graphene, which has high carrier mobility but zero bandgap limited its device application, and the transition-metal dichalcogenides (TMDs) that have the formula MX_2_ (M = Mo, W, Ge; X = S, Se, or Te), a class of 2D semiconductors have recently attracted remarkable scientific and technological interest for innovative devices. They exhibit a wide range of material properties, such as high carrier mobility for both electrons and holes, have a relatively large bandgap of 1.5–2.5 eV, have a tunable direct bandgap that ranges from 0.3 to 1.5 eV, have an ideal sub-threshold swing of ~ 60 mV/dec, an I_ON_/I_OFF_ ratio of 10^8^–10^9^, and high carrier mobility (200 cm^2^ V^−1^ s^−1^@ room temperature mobility for a single-layer MoS_2_ transistor with high-K dielectric and 1000 cm^2^ V^−1^ s^−1^@ 3 K temperature)^1–3^. Due to the weak van der Waals interface forces in graphene and TMDs materials, the p–n diode or the Schottky barriers (SBs) at the metal/TMDs interfaces have played an important role in electronic devices^4^. Besides, the electrical properties are hindered by the contact resistances rather than the intrinsic TMDs properties. The development of the TMDs with different bandgaps and work functions allow for the bandgap engineering of heterostructures that may possess new physical and electrical properties. Among TMDs materials, which include MoSe_2_, GeS, and GeSe, have been proposed as alternative 2D systems, and they have exhibited better performance in photodetectors. GeSe belongs to the layered IV–VI nanostructures since the p-type semiconductors with narrow bandgap (1.1–1.2 eV) are potential alternatives to the lead chalcogenides and open an avenue to fabricate highly efficient electronic and optoelectronic devices^5,6^. The MoSe_2_ from indirect (bulk crystal) to direct (monolayer) results in the bandgap increasing from 1.1 to 1.5 eV, which become direct in single atomic layers, and it makes them promising candidates in field-effect transistors (FETs), photovoltaic cells, light-emitting diodes (LEDs), and photodetectors. With the lower light response in photodetection/sensing, the bandgap tunability value for electrons or hole transfers between bulk TMDs materials down to the monolayer and slow electron transfer pathways is still greatly impeded. Consequently, consideration will still be given to matching the band alignment for electron or hole transfers between bulk materials for the development of photodetector creation.

By combing two semiconductors, the study of heterojunctions with the same lattice structures has become a hot topic in semiconductor technology. Recently, scientists have paid a great deal of attention to fabricating heterojunctions on graphene-like materials or heterojunctions by combining two semiconductors/semimetals, such as graphene-h-BN^7^, graphene-MoS_2_^8,9^, and graphene-MoSe_2_^10–12^. On the other hand, some heterojunctions are designed based on TMDs materials, which include [2D/2D] structures via either mechanical exfoliation or vapor deposition methods that include MoSe_2_/WSe_2_ heterojunction^13^, MoSe_2_/WS_2_ heterostructures^14^, MoS_2_/black phosphorus heterojunction^15^, p-type GaSe/n-type MoSe_2_^16^, [2D/1D] black phosphorus–zinc oxide nanomaterial heterojunction^17,18^, n-2D/p-oxide, and p-2D/n-oxide structures^17,19–21^, such as vertical MoSe_2_-MoO_x_^22^.

These novel semiconducting [2D/2D] TMDs/TMDs are now a primary focus of many researchers. Several TMDs based materials are under fabrication process to explore new physics and the next-generation photonic/optoelectronic devices. In the present study, we demonstrated the p-GeSe/n-MoSe_2_ heterojunction self-powered photodiodes. We observed good gate-tunable rectification characteristics of the p-GeSe/n-MoSe_2_ heterojunction p-n diode. The high ratification ratio of 1.4 × 10^4^ is obtained at V_bg_ = − 40 V. The photovoltaic behavior of the p-GeSe/n-MoSe_2_ heterojunction p-n diode at zero bias was investigated under various intensities of (53.3, 98.5, 123, and 139 mW/cm^2^) with NIR (850 nm) incident photons. The high responsivity (R = 465 mAW^−1^), the detectivity D of 7.3 × 10^9^ Jones, the normalized photocurrent to dark current ratio NPDR of 1.9 × 10^10^ W^−1^, the noise equivalent power NEP of 1.22 × 10^–13^ WHz^−1/2^, and the external quantum efficiency EQE of 670% were observed with a fast response time of 180 ms.The strong light-matter interaction in the device explicitly suggests that the p-GeSe/n-MoSe_2_ heterojunction p–n diode is a promising candidate for optoelectronics technologies.

## Experimentation

We prepared all the p-GeSe and the n-MoSe_2_ atomically thin flakes by peeling them from their parent bulk crystals using a scotch tape mechanical exfoliation technique, which is similar to the technique that is employed for the exfoliation of graphene^23,24^, and we transferred it onto a Si/SiO2 (300 nm) substrate using a transparent poly (dimethylsiloxane) (PDMS) stamp using an aligned dry transfer^25,26^. The multilayer p-GeSe and the n-MoSe_2_ flakes were identified using an optical microscope, and the multilayer n-MoSe_2_ was directly stacked on the top of the p-GeSe flake. Raman spectroscopy and atomic force microscopy (AFM) were also conducted. Electron beam lithography was used for the metal deposition of palladium/gold (Pd/Au:10/20 nm) and (Cr/Au:10/20 nm) onto the p-GeSe and the n-MoSe2, respectively. The lift-off processes were conducted to form electrodes on the multilayer p-GeSe and n-MoSe2 flakes. The electrical characterization at room temperature were exhibited using a Keithley 4200A-SCS parameter analyzer. The photovoltaic characteristics of the p-GeSe/n-MoSe_2_ heterojunction photodetector was performed using a continuous wave laser beam from a diode NIR laser (850 nm) that was directly illuminated onto the device.

## Result and discussions

**Figure 1:**
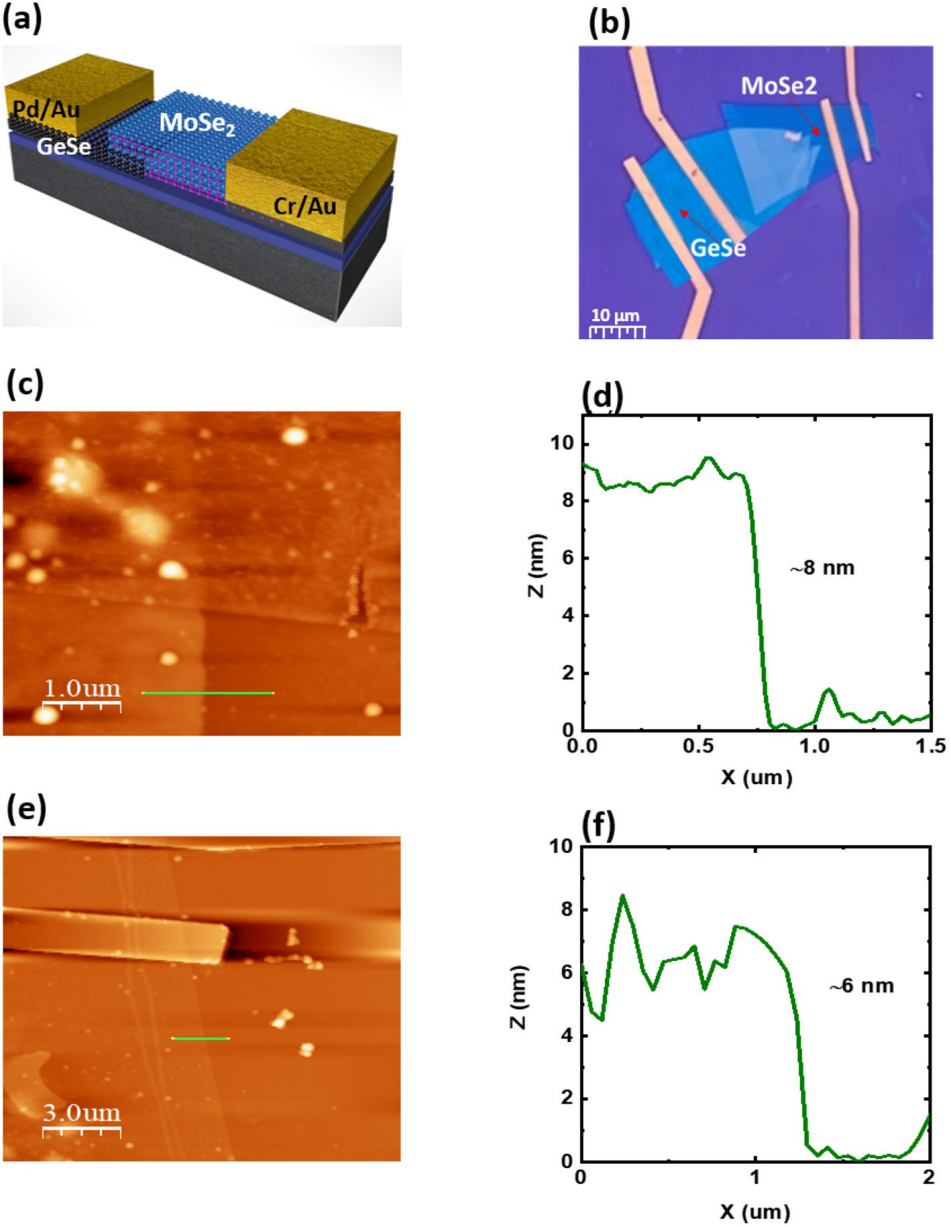
**(a)** A schematic view of the p-GeSe/n-MoSe_2_ heterojunction diode. **(b)** The optical microscope image of the device. **(c)** An AFM image of the p-GeSe on the SiO_2_/Si substrate. **(d)** The height profile from the AFM. **(e)** An AFM image of n-MoSe_2_ on the SiO_2_/Si substrate, and **(f)** their height profile.

**Figure 2:**
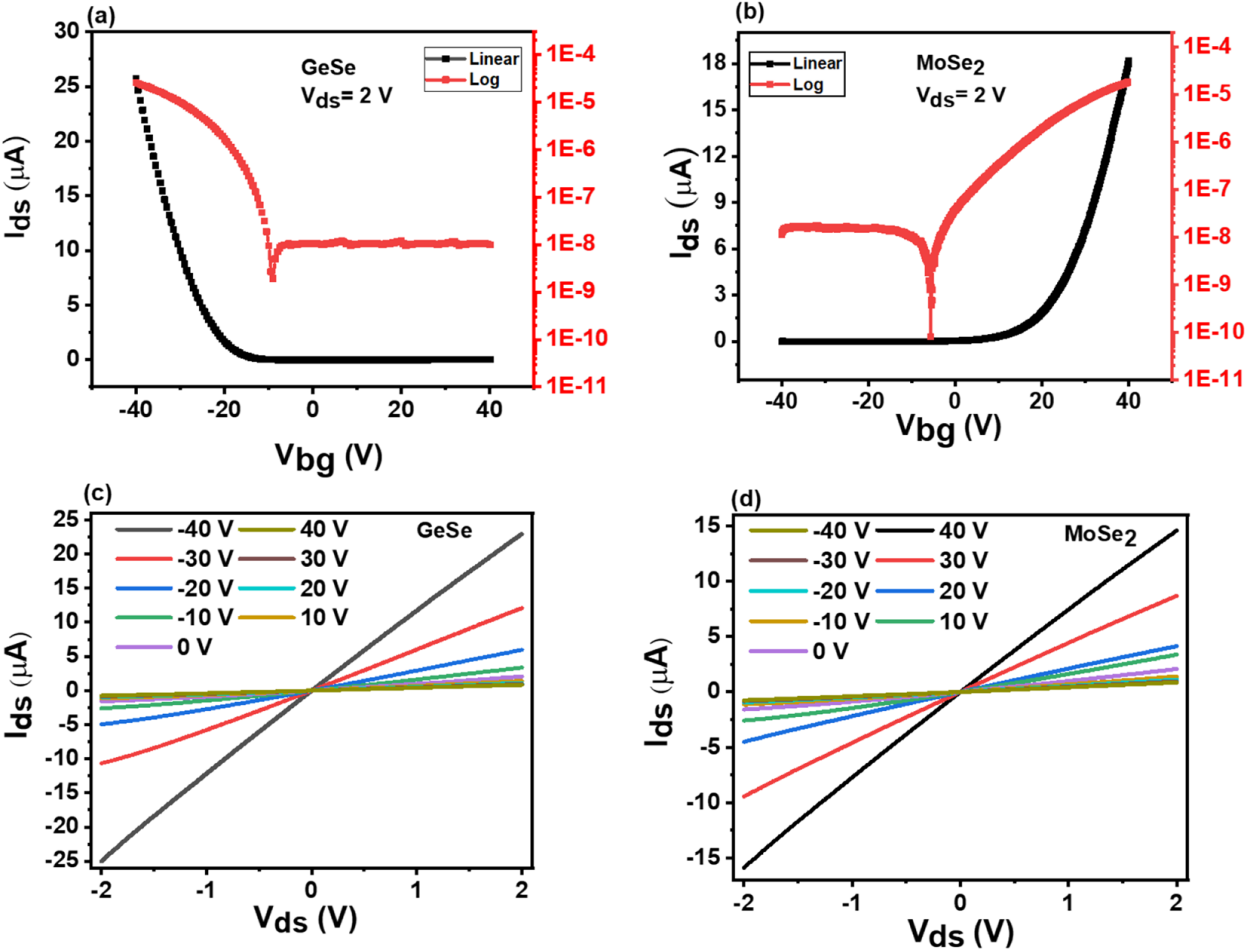
**(a)** The transfer characteristics of p-GeSe. **(b)** n-MoSe_2_. **(c)** The linear *I*_*ds*_*–V*_*ds*_ curves of p-GeSe shows ohmic behavior with Pd/Au, and **(d)** the linear *I*_*ds*_*–V*_*ds*_ curves of n-MoSe_2_ also show ohmic behavior with Cr/Au.

A schematic illustration of the demonstrated p-GeSe/n-MoSe_2_ heterostructure device is depicted in Fig. 1a. The optical image of the p-GeSe/n-MoSe_2_ heterostructure is shown in Fig. 1b. The atomically thin flakes of the p-GeSe and the n-MoSe_2_ were peeled from their parent bulk crystals using a scotch tape mechanical exfoliation technique, which was transferred on a 300 nm SiO_2_/Si substrate^27^. A few-layers of the n-MoSe_2_ were directly stacked on the top of the p-GeSe nanoflake by precisely determining their locations and an overlapping heterojunction region was formed. To clean the surface and optimize the charge carrier, the flakes of the p-GeSe and the n-MoSe_2_ were annealed at 200 °C for 1 h in an argon environment^28^. The Pd/Au (10/20 nm) and the Cr/Au (10/20 nm) optimum metal electrodes were then designed onto the p-GeSe and the n-MoSe2 flakes. Figure 1c–f shows the thickness of the p-GeSe (n-MoSe_2_), which is ~ 8 nm (~ 6 nm), and their height profiles that were measured by the atomic force microscopy (AFM) analysis. Raman spectroscopy was used to confirm the material p-type GeSe and the n-type MoSe_2_ shown in Figure S1(a). The p-GeSe/n-MoSe_2_ heterostructure device was electrically characterized at room temperature by applying drain to source voltage (*V*_*ds*_) and electrostatic back-gate voltage (*V*_*g*_). To validate the doping nature of the material GeSe and MoSe_2,_ the back-gate voltage (*V*_*g*_) was swept from − 40 to + 40 V at constant *V*_*ds*_ = 2 V, and the transfer characteristics revealed that the material GeSe (MoSe_2_) exhibited a p-type (n-type) nature with an ON/OFF ratio of 2.42 × 10^3^(1.28 × 10^3^), which is depicted in Fig. 2a, b. The semiconductor material and the metal interface could exhibit either ohmic behavior or rectifying behavior depending on the semiconductor and the metals working function values. Hence, we used optimum metal contacts for Pd (Φ ~ 5.6 eV)^29^ Cr (Φ ~ 4.5 eV) p-GeSe(n-MoSe_2_)^30,31^. Figure 2c shows ohmic behavior with a high work function of the Pd. To induce ohmic behavior between the metal and the n-MoSe_2_, we used the low work function of the Cr depicted in Fig. 2d. The field-effect carrier mobility (${\upmu }_{\mathrm{FE}}$) of the p-GeSe and the n-MoSe_2_ was calculated using the following equation^32–34^.

1 $${\mu }_{FE}= \frac{L}{W}(\frac{d{I}_{ds}}{d{V}_{bg}})\frac{1}{{C}_{bg}{V}_{ds}}$$

where W is the channel width, L is the channel length, C_bg_ is the gate capacitance (~ 115 aF/µm^2^) for the SiO_2_ substrate, and $(\frac{d{I}_{ds}}{d{V}_{bg}})$ is the slope of the transfer curve. The mobilities of the p-GeSe and the n-MoSe_2_ were estimated to be 110 cm^2^V^−1^ s^−1^ and 85 cm^2^V^−1^ s^−1^, respectively.

**Figure 3:**
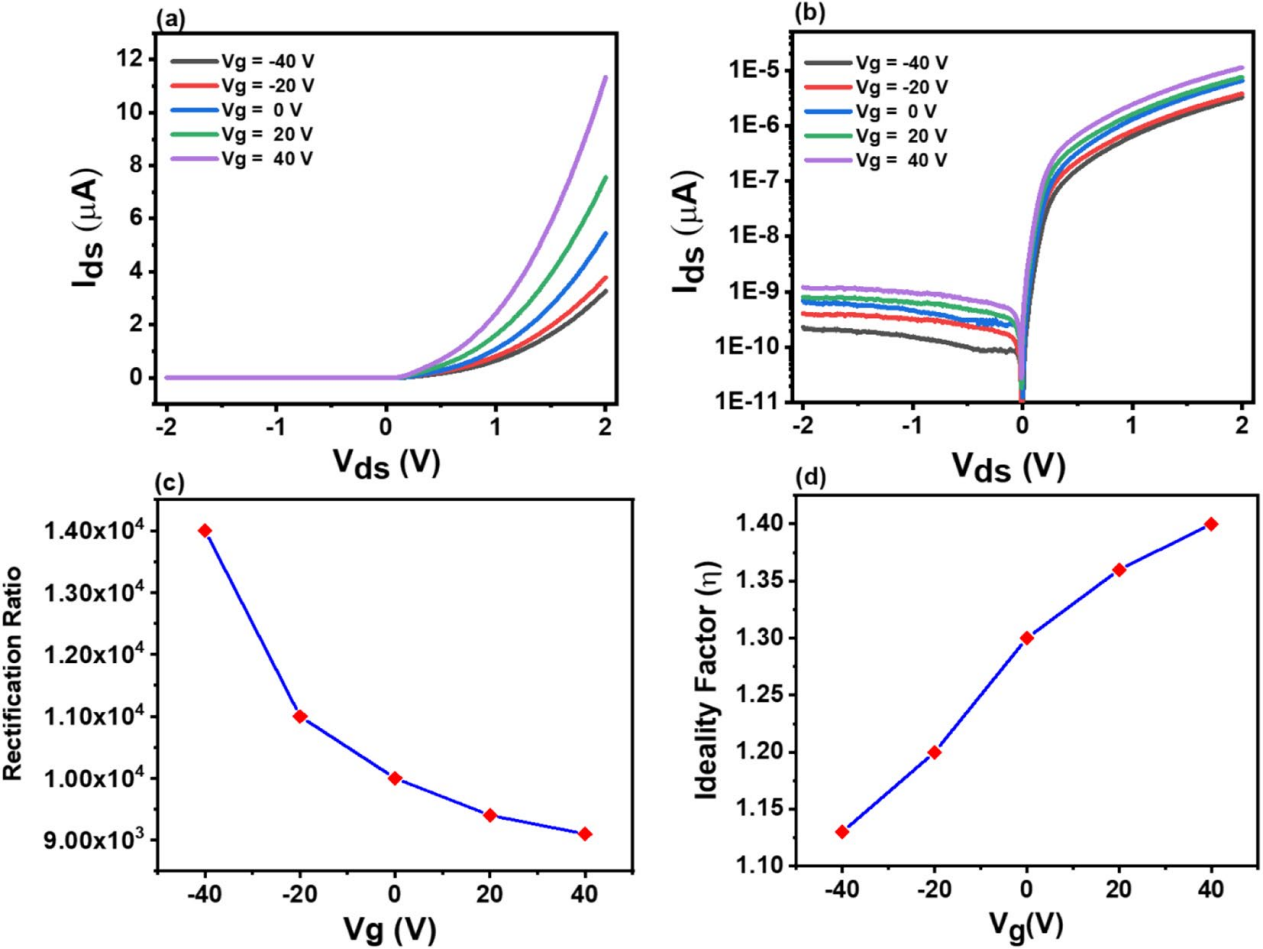
**(a)** The *I–V* characteristics of the p-GeSe/n-MoSe_2_ heterojunction diode at different gate voltages. **(b)** The corresponding semi-logarithmic plots of output characteristics. **(c)** The gate dependent rectification ratio of p-GeSe/n-MoSe_2_ heterojunction diode. **(d)** Gate dependent ideality factor of p-GeSe/n-MoSe_2_ heterojunction diode.

The gate-tunable electrical characteristics were also investigated. Figure 3a exhibits the gate dependent output characteristics of the p-GeSe/n-MoSe_2_ heterostructure diode, and Fig. 3b shows the same output curves in a corresponding logarithmic plot. It revealed that the rectifying behavior of the device is tuned by the electrostatic gate-voltage. The forward bias rectifying current increases as the gate voltages (*V*_*g*_) increased from *− V*_*g*_ to + *V*_*g*_, the electrons are attracted to the interface between GeSe and SiO_2_ to form accumulation layer results the Fermi level of GeSe moves towards the conduction band and lowering potential barrier height results in decreasing rectification current attributed to electrostatic doping of electrons. Moreover, we investigated the rectification ratio, which is defined as the ratio of the forward current over the reverse current, *I*_*f*_*/I*_*r*_*,* up to 1.4 × 10^4^ at *V*_*g*_ = − 40 V. We found that at a positive gate voltage of *V*_*g*_ =  + 40 V, both the reverse and forward currents increase concurrently, which suppress the rectification as a result. In anticipation of the negative gate voltage, the reverse current is constrained to increase the rectification in the p-GeSe/n-MoSe_2_ heterostructure diode, which is depicted in Fig. 3c. Additionally, we estimated the ideality factor to confirm the performance of the rectifying behavior of the p-GeSe/n-MoSe_2_ heterojunction diode using the thermionic emission theory^4,35^.

2 $${I}_{D}={I}_{S}\left[\mathrm{exp}\left(\frac{qV}{n{K}_{B }T}\right)-1\right]$$

where ${I}_{S}$ is the reverse bias saturation current,$n$ is the ideality factor, q is the elementary charge, T is the temperature, and ${K}_{B}$ is Boltzmann’s constant. After the interpretation above, the equation becomes

3 $$\mathit{ln}\left({I}_{D}\right)=\mathit{ln}\left({I}_{S}\right)+ \left(\frac{q}{n{K}_{B }T}\right)V$$

The ideality factor (n) can be obtained via the following equation.

4 $$n={\left(\frac{q}{{K}_{B }T}\right)}\bigg/{\left(\frac{{\mathrm{d}}V}{{\mathrm{dln}}I_D}\right)}$$

**Figure 4:**
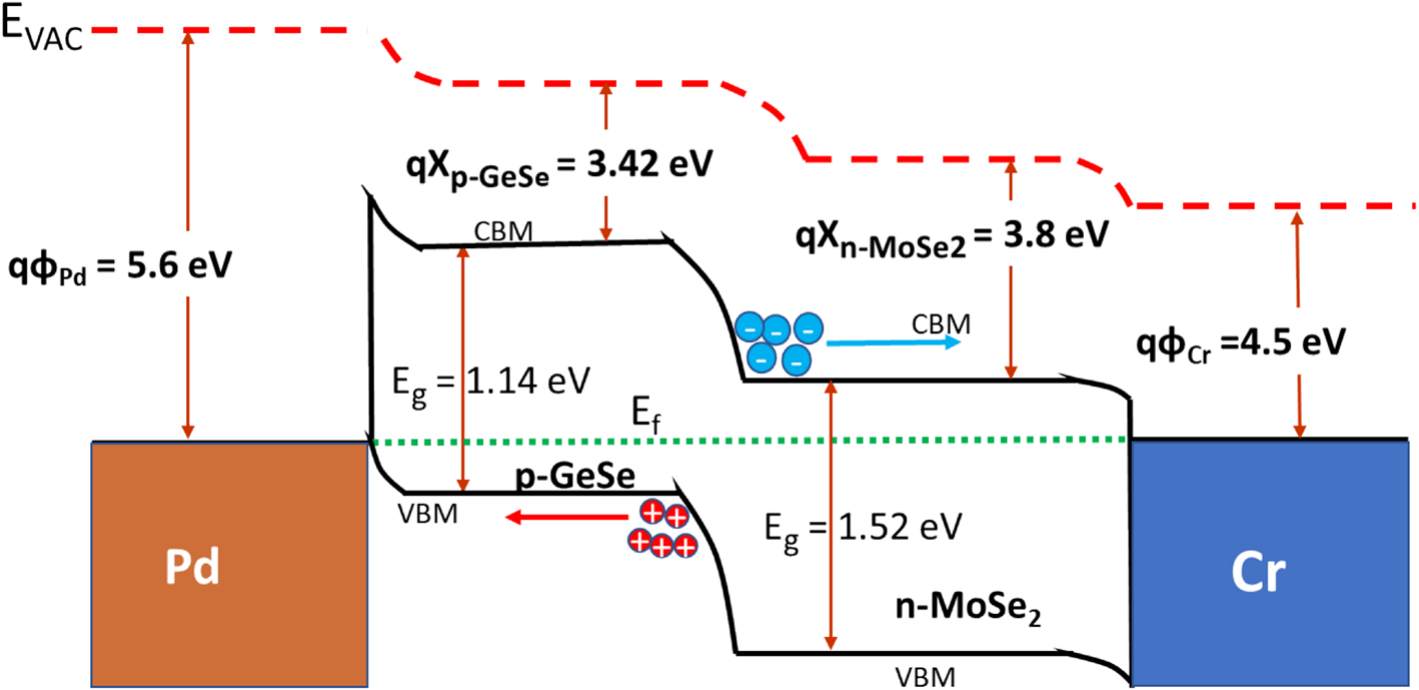
The band diagram of p-GeSe/n-MoSe_2_ heterojunction diode with metal electrodes.

Figure 3d illustrates the ideality factor function of the gate voltage, the ideality factor of 1.1 is obtained at *V*_*g*_ = − 40 V*,* which is close to the ideal diode value (η = 1). The relative degrading of the gate tunable ideality factor is attributed to the surface carrier recombination at the interface of the p-GeSe/n-MoSe_2_ diode, which results in a decrease in the electric field^21^. The variation in the electron affinity and the bandgap between the monolayers generates an atomically sharp hetero-interface, and the interface band alignment of the p-GeSe/n- MoSe_2_ heterostructure is predicted to be a type II band alignment, which is shown in Fig. 4.

**Figure 5:**
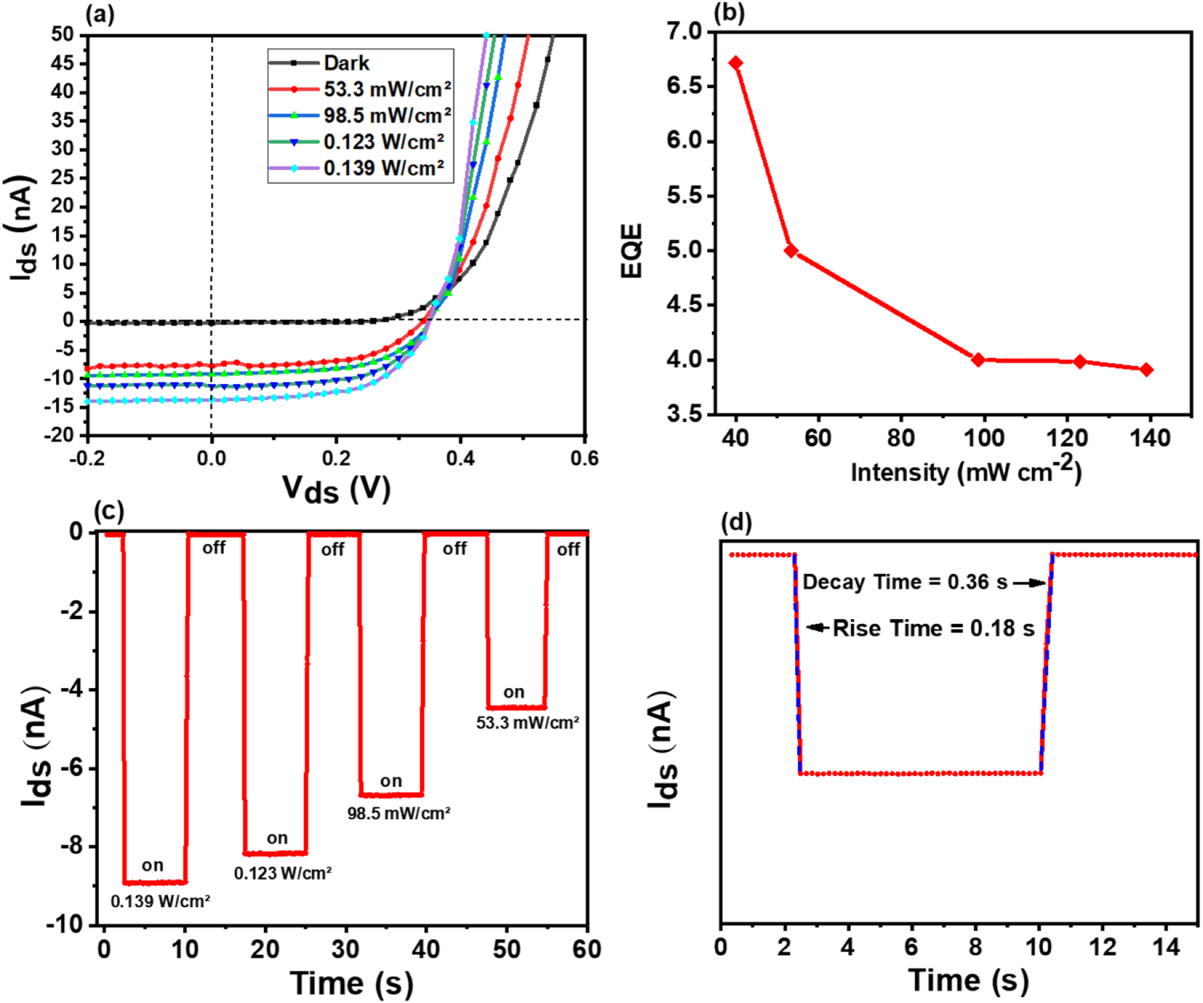
**(a)** The *I–V* characteristics of p-GeSe/n-MoSe_2_ heterojunction diode under dark and variable intensities. **(b)** The external quantum efficiency EQE function of incident power. **(c)** The time-dependent photoresponse of p-GeSe/n-MoSe_2_ heterojunction diode under illuminations with different laser light (@850 nm) intensity at V_ds_ = 0 V. **(d)** The rise time and decay time.

Furthermore, we investigated the self-powered photovoltaic characteristics of a p-GeSe/n-MoSe_2_ heterostructure device. The self-powered photodetectors are devices that can separate photoexcited carriers by the built-in electrical field at the junctions without any external power source. On this principle, the p-n junctions can be established for the photovoltaics^36,37^. We used an NIR (850 nm) laser with various illumination power intensities (53.3, 98.5, 123, and 139 mW/cm^2^) to measure the photocurrent generated from the photodiode that was based on the p-GeSe/n-MoSe_2_ heterojunction. A strong photoresponse was observed in the p-GeSe/n-MoSe_2_ junction region, which showed that a continuous charge separation occurred at the junction. Figure 5a presents the *I*_*ds*_*–V*_*ds*_ curves of the p-GeSe/n-MoSe_2_ heterojunction in dark and under photon irradiation with wavelength of 850 nm at zero bias with a constant gate voltage (*V*_*g*_ = 0). The *I*_*ds*_*–V*_*ds*_ curves are shifted down under the irradiation of light, which revealed that the device can be developed for self-powered photovoltaic energy conversion under the action of open-circuit voltage (V_oc_). We investigated an open-circuit voltage (V_oc_) of 0.349 V and a short-circuit current (I_sc_) of 14.5 nA for the 139 mW cm^−2^ light intensity. The external quantum efficiency (EQE) was investigated by using the following formula.

5 $$EQE=\frac{hc}{e\lambda }R$$

where λ is the incident light wavelength, *h* is the planks constant, and c is the velocity of light. We obtained a value for EQE of 670% in the p-GeSe/n-MoSe_2_ diode. The power intensity-dependent EQE is depicted in Fig. 5b. Additionally, we also characterized the transient photoresponse of the device. The dynamic photoresponse rise and fall time of the p-GeSe/n-MoSe_2_ diode was observed under an NIR laser light irradiation with a wavelength (λ) of 850 nm at various power intensities, which is shown in Fig. 5c. The rise time is the τ_r_, the time it takes by the device to reach 90% from 10% and the fall time is τ_f_, the time it takes by the device to decay from 90 to 10%^36,38,39^. We found a rise time of 180 ms and a fall time of 360 ms, which are shown in Fig. 5d. The response time of the device is not as fast as we expected, which may be due to the charge carrier trapping and the longer charge dissociation time^40–42^.

**Figure 6:**
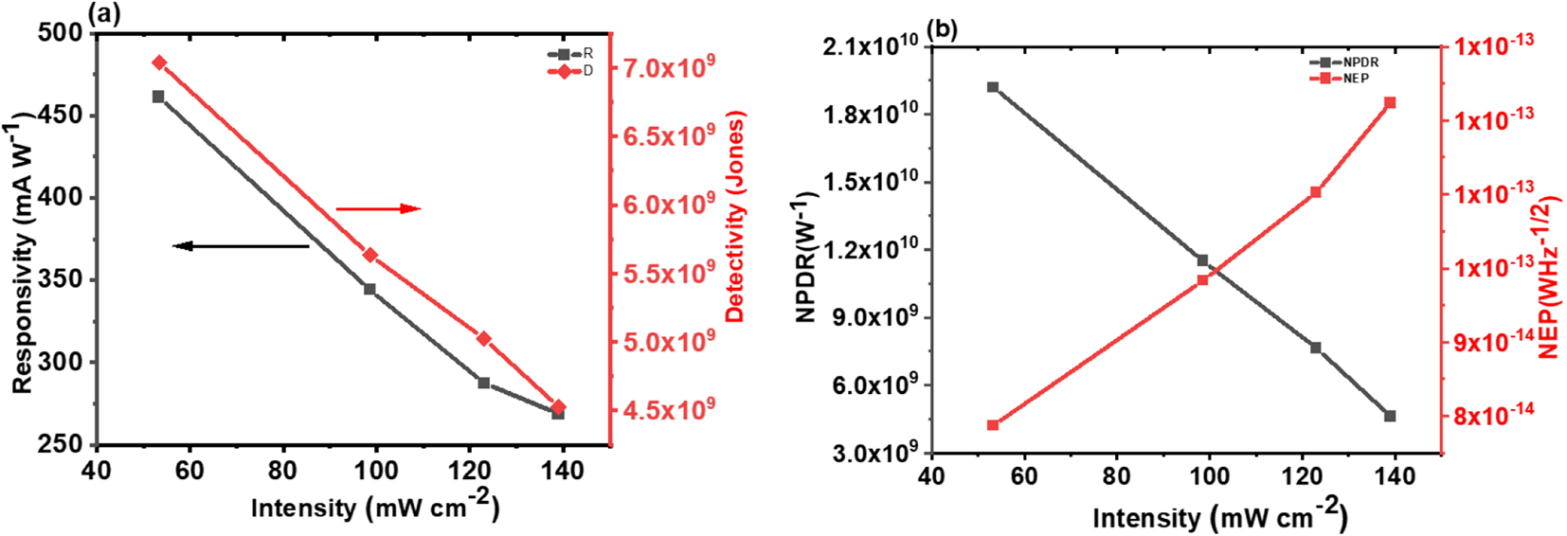
**(a)** The responsivity, R (mA W^−1^), and the detectivity, D (Jones) function of power intensities **(b)** the normalized photocurrent to dark current ratio NPDR (W^−1^) and the noise equivalent power NEP (W Hz^−1/2^) function of power intensities.

Moreover, in order to evaluate the performance of the device, several important figures of merits were calculated. For example, responsivity (R), detectivity (D), the normalized photocurrent to a dark current ratio (NPDR), and the noise equivalent power (NEP) with variation of incident light power intensities were calculated. The responsivity ($\mathrm{R}= {\mathrm{J_p}}/{P_{in}}$), where J_p_ is the photocurrent density and P_in_ is input power per area, and the detectivity ($D={R}/{\sqrt{2qJ_d}}$), where q is the elementary charge and J_d_ is the dark current density, are significant facets of the photo detector^36,38,39^ , which is shown in Fig. 6a. The greater value of responsivity is attributed to the higher photocurrent^43^. Similarly, the device that has a lower dark current provides a higher detectivity. Thus, the greater values of both R and D are important aspects of an efficient photodetector^37,43,44^. We obtained a high responsivity of R = 465 mAW^−1^ and detectivity of D = 7.3 × 10^9^ Jones.

Figure 6b shows the intensity-dependent normalized photocurrent to dark current ratio. The *NPDR* = *R/I*_*d*_, where *R* is the responsivity, *I*_*d*_ is the dark current, and the noise equivalent power (NEP = *1/*($NPDR\sqrt{2q/I_d}$).We investigated the values of NPDR of 1.9 × 10^10^ W^−1^ and NEP of 1.22 × 10^–13^ WHz^−1/2^ under the power intensity of 139 mW cm^−2^. The NEP revealed that the photodetector, which is based on the p-GeSe/n-MoSe_2_ heterostructure, has the capability of detecting power as low as 10^–13^. Additionally, we characterized the spectral responsivity of the p-GeSe/n-MoSe2 heterojunction. The device was subjected to constant illuminating power of 53 mW cm^-2^ with wavelength ranging from 220 to 850 nm. Figure S1c shows a sharp increase of the spectral response on the short wavelength side is reasonably due to more photon energy absorbed by the device, attributed to more electrons and holes generation under larger photons energy. Table S1 in supplementary information shows the comparative investigated photoresponse and sensitivity of our device based on p-GeSe/n-MoSe_2_ heterojunction, which is much higher than the previously reported values. The strong light-matter interaction in the device explicitly suggests that the p-GeSe/n-MoSe_2_ heterojunction p–n diode is a promising candidate for optoelectronics technologies.

## Conclusions

In summary, we demonstrate a p-GeSe/MoSe_2_ based multifunctional HJ p–n diode. The diode explicitly exhibits gate tunable high rectification of ~ 1 × 10^4^ at negative gate bias (Vg = − 40 V). The introduction of the ohmic contacts reveals that the rectification behavior of a p–n diode is solely attributed to the sharp interface between metal contacts and GeSe/MoSe_2_. Our device shows high photoresponse at an NIR (850 nm). The high responsivity of 465 mAW^−1^, the excellent EQE (670%), the fast rise time of 180 ms, and the decay time of 360 ms were obtained. The device also shows detectivity D of 7.3 × 10^9^ Jones, a normalized photocurrent to dark current ratio NPDR of 1.9 × 10^10^ W^−1^, and a noise equivalent power NEP of 1.22 × 10^–13^ WHz^-1/2^.The NEP revealed that the photodetector, which is based on the p-GeSe/n-MoSe_2_ heterostructure, has the capability of detecting power as low as 10^–13^. These results suggest that p-GeSe/MoSe_2_ based multifunctional heterojunction p–n diode may have great potential for electronics and optoelectronics applications as high-performance self-powered photodetectors.

## Supplementary Information

Supplementary Information.
