# Supplementary material for: NIR self-powered photodetection and gate tunable rectification behavior in 2D GeSe/MoSe2 heterojunction diode
Source: Sci Rep. 2021 Feb 11;11:3688. doi: 10.1038/s41598-021-83187-z (PMC7878902; doi:10.1038/s41598-021-83187-z)
Supplement: Supplementary file 1 — Supplementary Information. [file 41598_2021_83187_MOESM1_ESM.docx]

**NIR self-powered photodetection and gate tunable rectification behavior in 2D GeSe/MoSe_2_ heterojunction diode**

Muhammad Hussain^1^, Syed Hassan Abbas Jaffery^1^, Asif Ali^1^, Dinh Nguyen Cong^1^, Sikandar Aftab^2^, Muhammad Riaz^1^, Sohail Abbas^3^, Sajjad Hussain^1^, Yongho Seo^1^ & Jongwan Jung^1*^

^1^ Department of Nanotechnology and Advanced Materials Engineering, and HMC, Sejong University, 05006, South Korea.

^2^Department of Engineering ,Simon Faster University, Burnaby, Canada.

^3^Faculty of Engineering and Applied Sciences, Ripah International University ,Islamabad Pakistan.

*Corresponding author: [jwjung@sejong.ac.kr](mailto:jwjung@sejong.ac.kr)

**Supplementary Information**


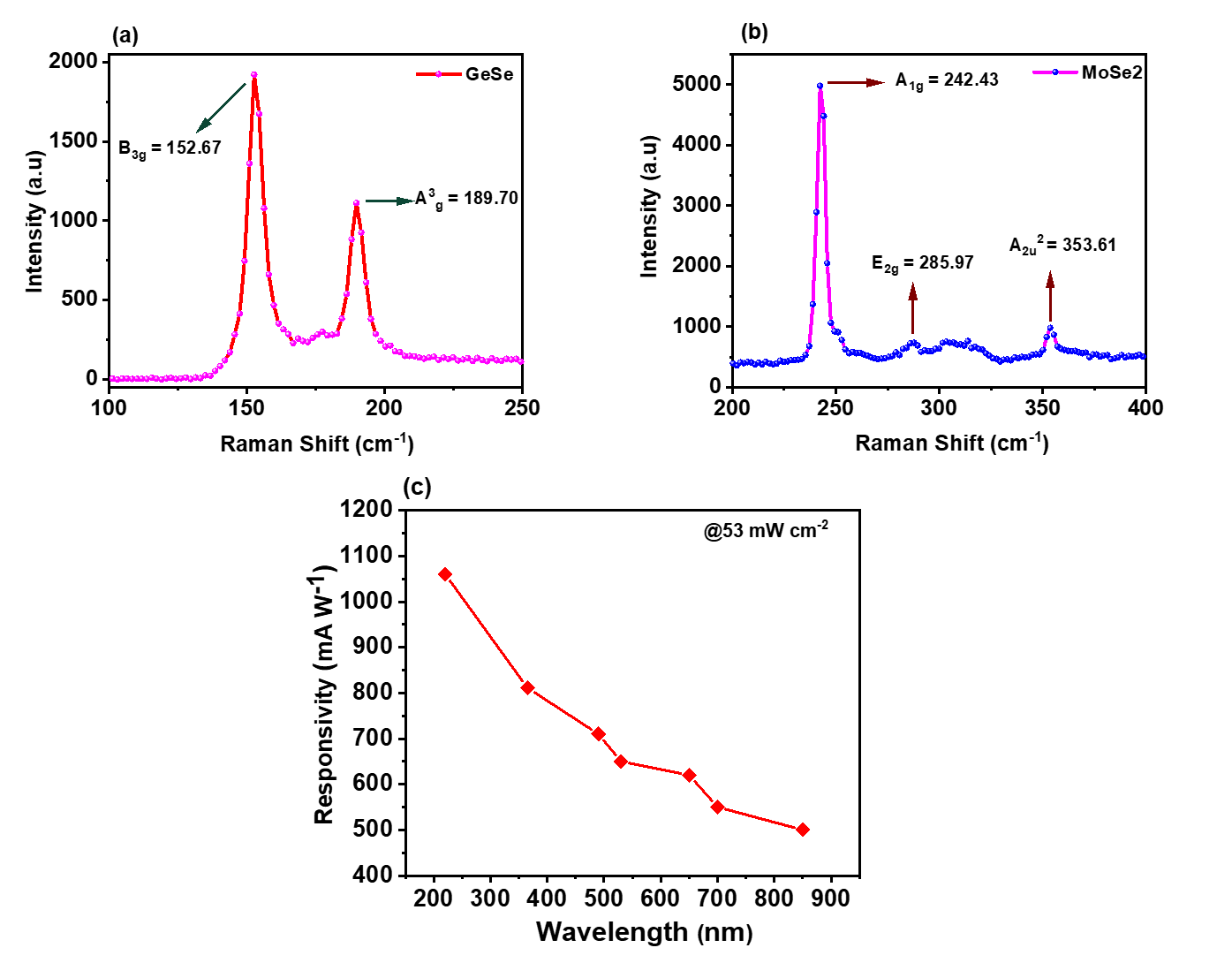


**Figure S1.** The Raman spectra of the p-GeSe flakes and (b) the n-MoSe2 flakes.(c) spectral photoresponse of p-n diode heterojunction ranging from 220 nm to 850 nm with the constant illuminating power of 53 mW cm-2was characterized.

The Raman spectra of GeSe and MoSe_2_ are presented in Figure S1a and b, respectively. The Raman characteristics are studied under 514.5 nm laser excitation with an exposure time of 10s. The Raman peaks of GeSe are observed at 152.67 cm^-1^ (B_3g_ out‐of‐plane mode) and 189.70 cm^-1^ (A^3^_g_ in‐plane mode), while the peaks of MoSe_2_ are observed at 242.34 cm^-1^ (A_1g_ out-of-plane), 285.97 cm^-1^ (E_2g_ in-plane optical mode) and 353.61 cm^-1^ (interlayer interaction). The presence of Raman peak at 353.61 cm^-1^ (interlayer interaction) confirms the few-layer MoSe2 flake. These data are consistent with the previous results. ^1-4^

**Table S1:**

| p-n Heterojunction | Thickness  (nm) | Ideality factor | Bias Voltage (V) | Wavelength (nm) | Detectivity (Jones) | Responsivity (mA/W) | NPDR (W^-1^) | NEP (WHz^-1/2^) | Ref |
| --- | --- | --- | --- | --- | --- | --- | --- | --- | --- |
| GeSe/MoSe_2_ | **8/6** | **1.12** | **0** | **850** | **7.3×10^9^** | **475** | **1.9×10^10^** | **10^-13^** | **This work** |
| WSe_2_/MoS_2_ | **0.8/0.8** | **-** | **0** | **532** | **-** | **10** | **-** | **-** | **^5^** |
| P/MoS_2_ | **11/0.9** | **-** | **-2** | **633** | **-** | **418** | **-** | **-** | **^6^** |
| WSe_2_/MoS_2_ | **25/18** | **1.5** | **0** | **532** | **-** | **170** | **-** | **-** | **^7^** |
| ReS_2_/ReSe_2_ | **64/48** | **-** | **1** | **550** | **-** | **21.07** | **-** | **-** | **^8^** |
| BP/ReS_2_ | **5/12** | **1.04** | **1** | **532** | **-** | **8** | **-** | **-** | **^9^** |
| MoS_2_ /WSe_2_ | **0.65/0.7** | **-** | **-2** | **532** | **-** | **11** | **-** | **-** | **^10^** |


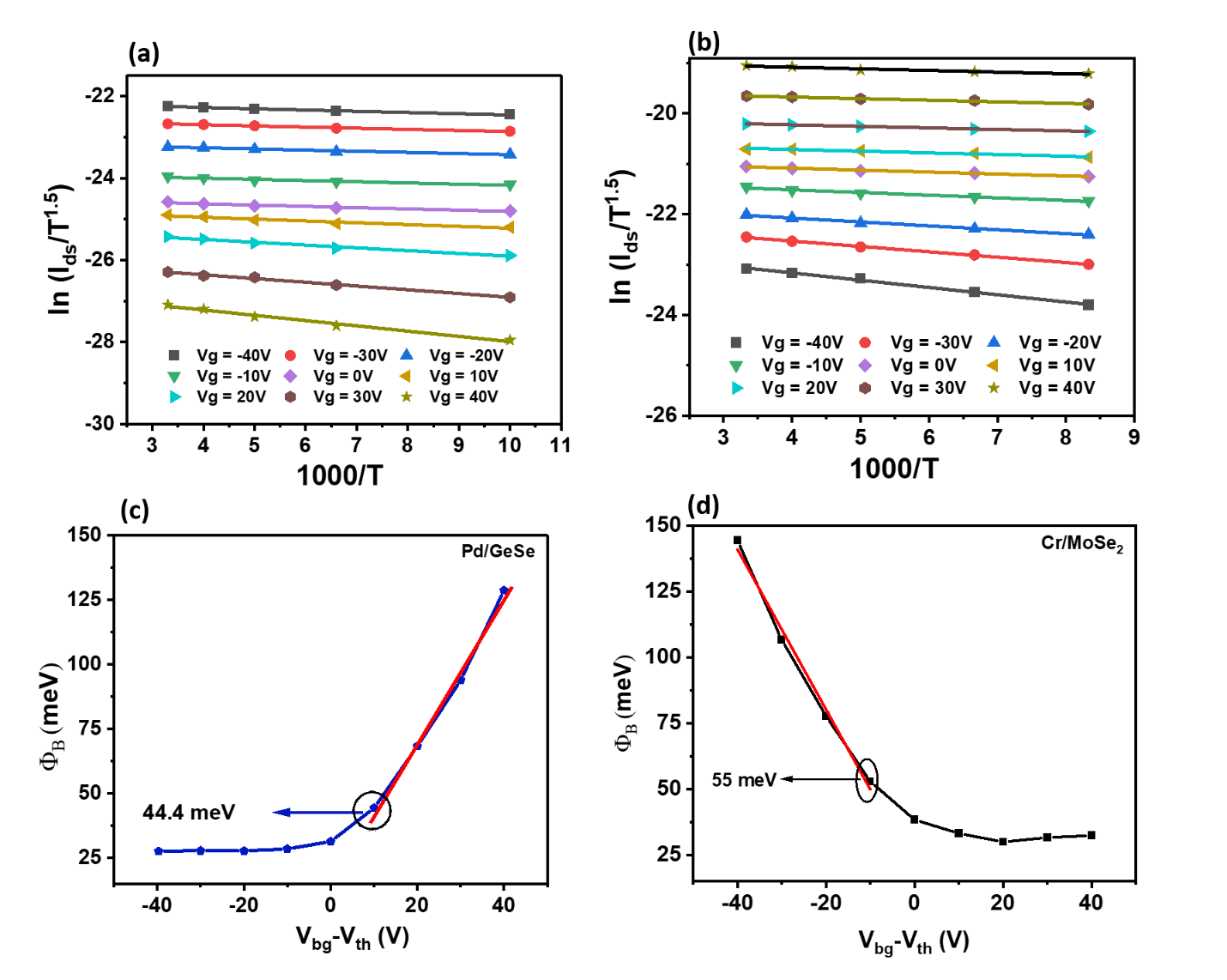


**Figure S2.** (a) Typical Richardson’s plot ln (I_ds_/T^1.5^) versus 1000/T for (a) Pd/GeSe and (b) for Cr/MoSe_2_ ,respectively. Corresponding barrier height of (c) Pd/GeSe and (d) Cr/MoSe_2_ contacts as a function of *Vbg-Vth.*

To extract the barrier height (BH) of Pd (ϕ = 5.6 eV) and Cr (ϕ = 4.5 eV), with p-GeSe and n-MoSe_2_ ,respectively, we measured temperature-dependent transfer characteristics. According to thermionic emission theory, we used the following equation^11-16^,

$I_{\mathrm{ds}}=A_{device}A^{*} T^{\alpha}\exp\left[ -\frac{q}{k_{B}T} \left( \Phi_{B}- \frac{V_{\mathrm{ds}}}{n} \right) \right]$ (1)

where $A_{device}{, A}^{*}$ are an active area of p-n diode and Richardson constant, q is the elementary charge of the electron, T is the temperature K_B_ is Boltzmann’s constant, V_ds_ the source-drain voltage, α is an exponent equal to 2 for bulk SCs and 3/2 for 2D SCs respectively and n is the ideality factor. We drew the Arrhenius plot of the device at various gate voltages for both metals Pd and Cr showed in Figure S2 a and b, respectively. The slope of ln (I_ds_/T^2^) versus 1000/T gives the BH for Pd and Cr with p-GeSe and n-MoSe_2_,respectively, as shown in Figure 3c and 3d. We extracted the value of BH of Pd with p-GeSe and Cr with n-MoSe_2_ of 44.4 meV and 55 meV , respectively.

Furthermore, we have characterized the time-dependent photoresponse at different gate voltages. Without any external bias voltage and light irradiation, the p-GeSe/n-MoSe_2_ heterostructure diode reached thermal equilibrium condition by Fermi level pining results potential barrier height formation at p-GeSe/n-MoSe_2_ heterostructure interface. At negative gate voltage, the Fermi level of p-GeSe shifted towards valence band and increase the potential barrier height at the interface of p-GeSe/n-MoSe_2_ heterostructure diode, gives high internal built-in potential, results greater photovoltaic behavior without external power. While at V_g_> 0 the Fermi level moves towards conduction band and lowering potential barrier height results lowering internal built-in potential causes smaller photovoltaic current under the illumination of NIR wavelength of 850 nm with power of 53.3 mWcm^-2^ V_ds_ =0 V.

**
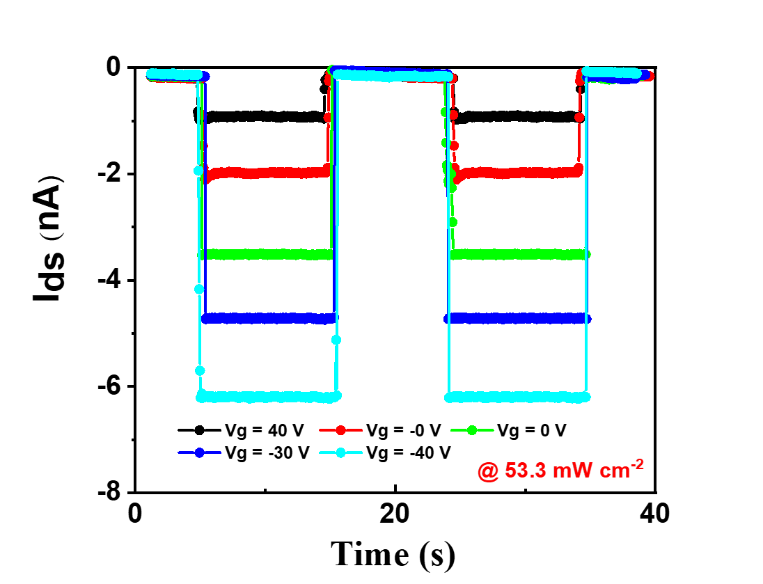
**

**Figure S3** Gate tunable transient photoresponse p-GeSe/n-MoSe_2_ heterojunction diode *V_g_* -40 V to +40 V with constant illumination power of 53.3 mW cm^-2^.

**
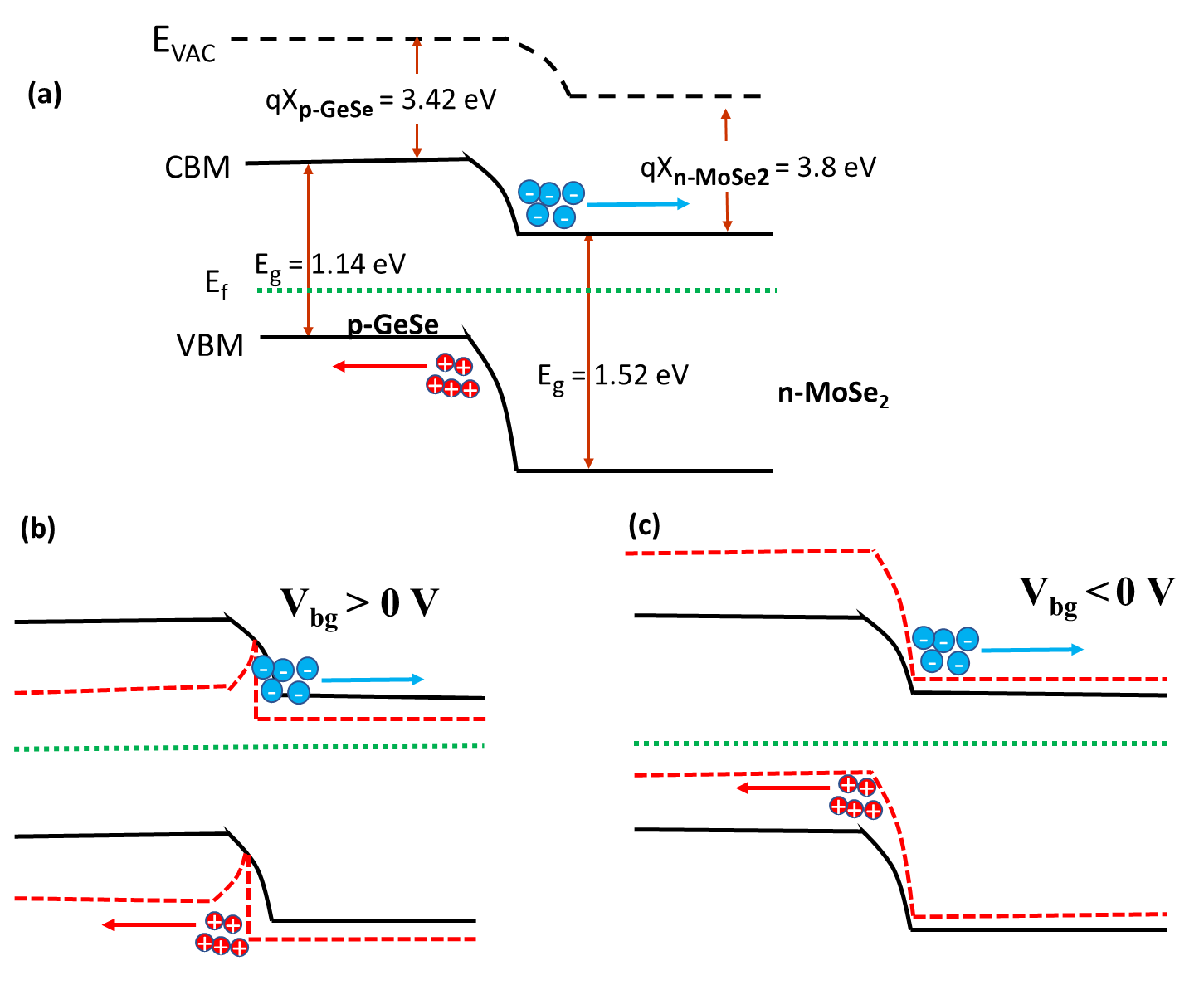
**

**Figure S4 (a)** Band diagram of p-GeSe/n-MoSe_2_ heterojunction diode at *V_g_ =* 0 V (b) *V_g_ >*0 V and (c) *V_g_ < 0* V. At negative back-gate voltage ,the Fermi level of GeSe shifted towards valence band and increase the potential barrier height at the interface of p-GeSe/n-MoSe_2_ heterostructure diode ,results high rectification current . While at V_g_> 0 the Fermi level moves towards the conduction band and lowering potential barrier height results decreasing rectification current.

we have estimated trapped states for both GeSe and MoSe_2_ by using temperature dependent *I_ds_-V_ds_* curves, plotting in logarithmic scale. The *I*ds–*V*ds curves at different temperatures intersect each other at a specific *V*ds value, as seen in figures (a) and (b). This significant value of *V*ds corresponds to *V*c and can be denoted by the following equation^17^.

$V_{c}=\frac{qN_{t}d^{2}}{2\epsilon_{0}\epsilon_{r}}$ 2

Where *N*_t_ is the density of trap states, *d* is the thickness of channel, $\epsilon$_o_ is the permittivity of free space, and $\epsilon$_r_ is the dielectric constant. The density of trap states *N_t_* for GeSe and MoSe_2_ were estimated of 7.6 ×10^12^ cm^-3^ and 1.6 ×10^12^ cm^-3^ respectively.


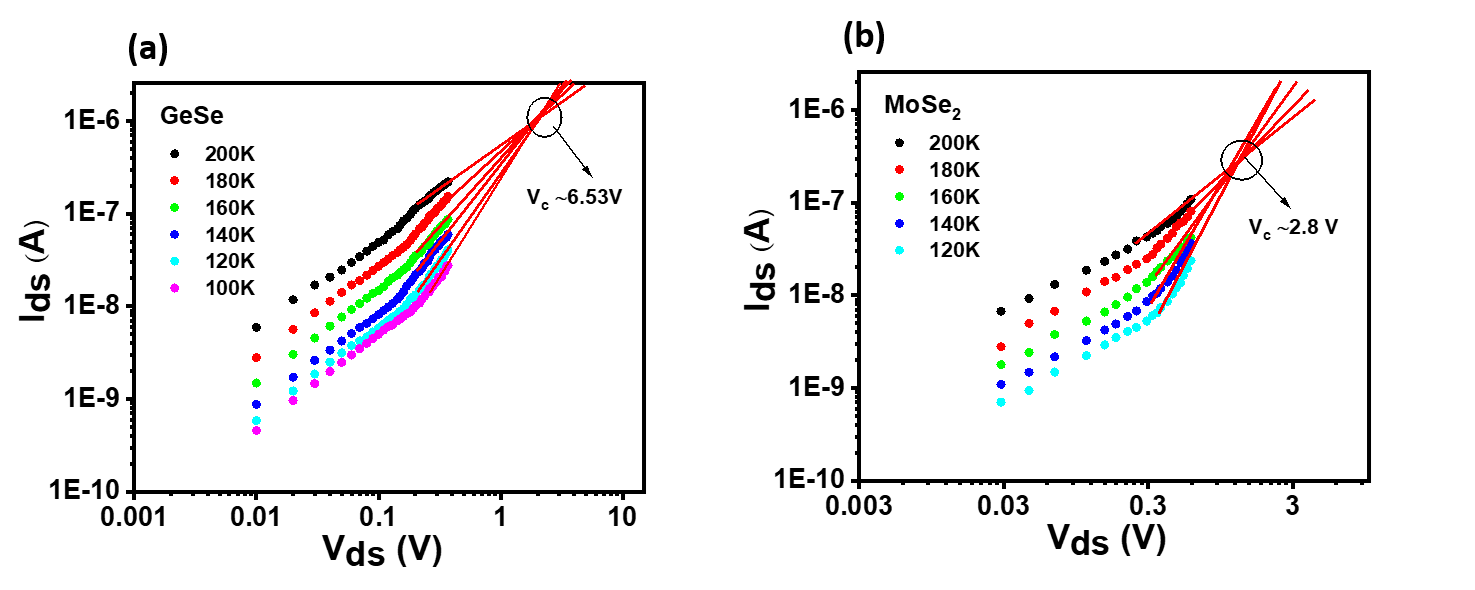


**Figure S5 (a).**Temperature dependent *I_ds_-V_ds_* output characteristics of GeSe device and **(b)** MoSe_2_ device.


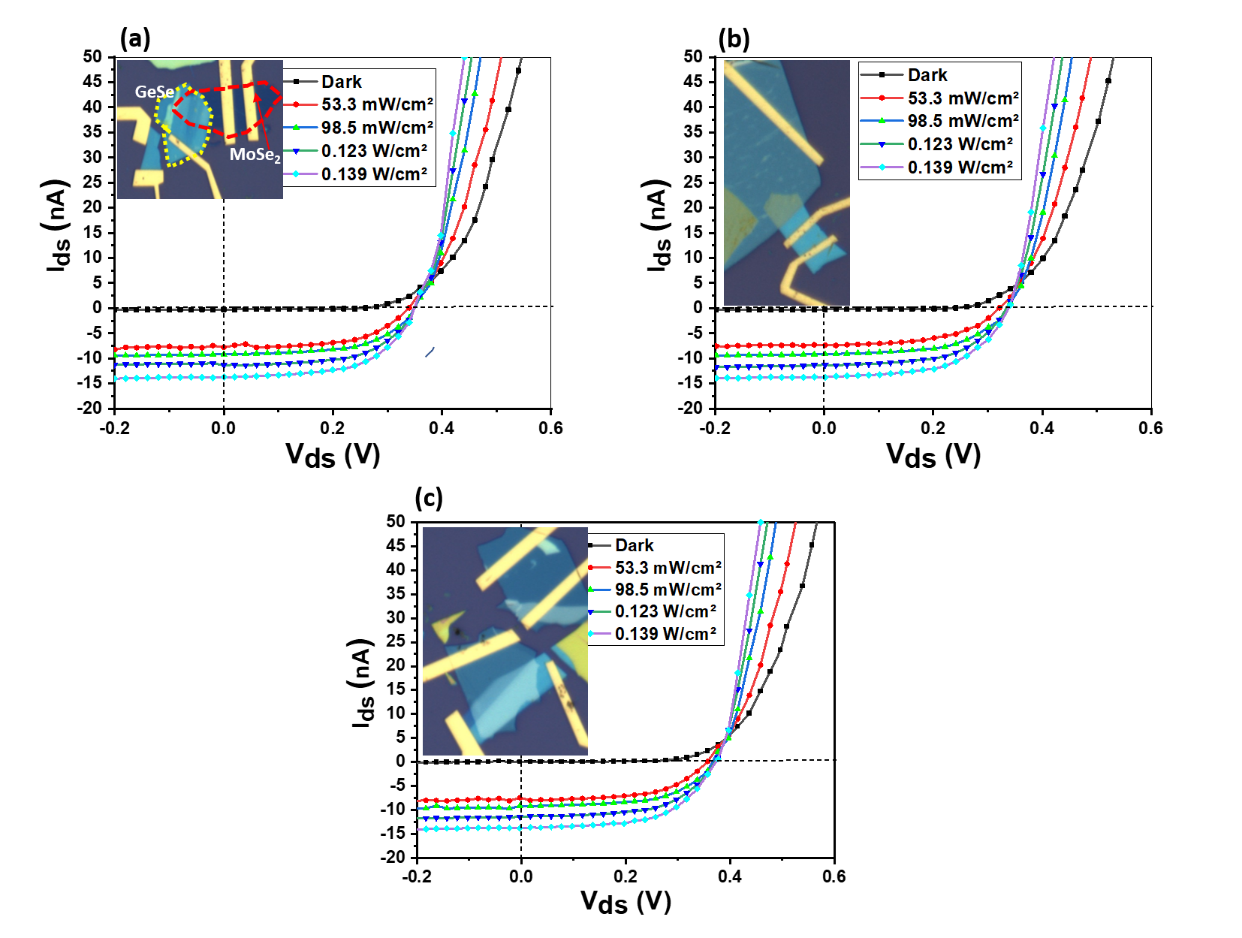


**Figure S6 .** The *I-V* characteristics of p-GeSe/n-MoSe_2_ heterojunction diode under dark and variable intensities. (a) Device 1 (b)Device 2 (c) Device 3

To validate the bang gap energies of GeSe and MoSe_2_ the photoluminescence (PL) spectrum characterization was carried out, PL spectrum of GeSe and MoSe_2_ samples are measured from visible to near infrared wavelengths, Illustrated in Figure S7 a & b. PL main peaks in GeSe and MoSe_2_  were found at 1060 nm and 805 nm respectively. Thus, we obtain the bang gap energies of GeSe and MoSe_2_  about 1.17 eV and 1.54 eV respectively.


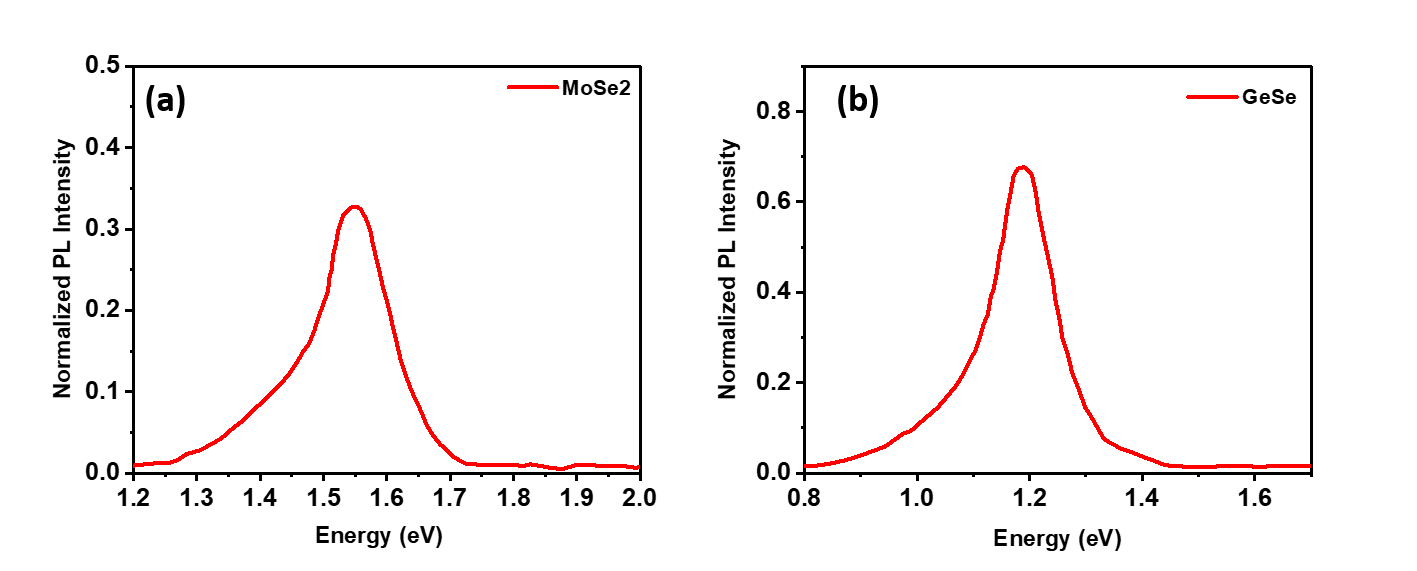


Figure .S7 Photoluminescence spectrum of the (a) MoSe_2_ and (b) GeSe.

Kelvin probe force microscopy (abbreviated as KPFM) is a method based on atomic force microscopy (AFM) used to analyze the electronic properties of nanoscale materials. KPFM quantifies the contact potential difference (CPD) between an AFM prob tip and the material surface by electrostatic force ^18-20^. The electronic properties (built-in potential, depletion layer and doping concentration) of GeSe/MoSe_2_ p-n heterostructure have been analyzed by KPFM. The local contact potential difference (CPD) between the local area of GeSe or MoSe_2_ and AFM tip can be stated as :

${CPD}_{GsSe}=W_{tip}-W_{GeSe}$ 3

${CPD}_{MoSe2}=W_{tip}-W_{MoSe2}$ 4

Whereas, W_tip_ work functions of the AFM tip , W_GeSe_ and W_MoSe2_ are the work functions of the AFM tip, GeSe and MoSe_2,_ respectively. Thus ,we have obtained the change in V_CPD_ across the the junction ~316 mV, reflects the built-in potential of GeSe /MoSe_2_ heterojunction junction shown in **Figure S8 b** . And the depletion width of 0.5 µm was obtained. Moreover, in order to calculate the doping concentration of GeSe and MoSe_2_ ,we obtain the built-in electric field distribution by differentiating the built-in potential ,the fitted built-in electric field distribution curve (data not mentioned) can give the doping concentration *N_A_* _­­_in GeSe around 2.4x10^14^ cm^‑2^ and *N_D_* in MoSe_2_ around 1.6x10^14^ cm^‑2^ .


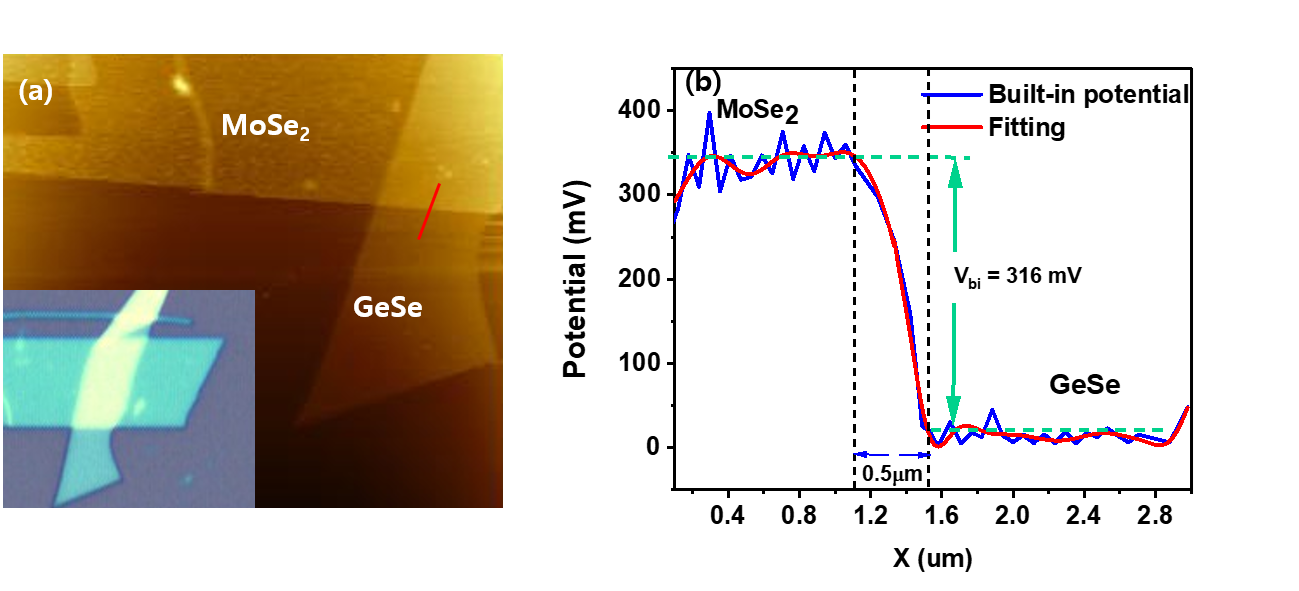


Figure S8 a) AFM image of p-GeSe/n-MoSe_2_ heterostructure . b) The corresponding built-in potential distribution profile.

1. Yang, M.; Cheng, X.; Li, Y.; Ren, Y.; Liu, M.; Qi, Z. *Applied Physics Letters* **2017,** 110, (9), 093108.

2. Nam, D.; Lee, J.-U.; Cheong, H. *Scientific Reports* **2015,** 5, (1), 17113.

3. Zhao, Y.; Lee, H.; Choi, W.; Fei, W.; Lee, C. J. *RSC Advances* **2017,** 7, (45), 27969-27973.

4. Zhao, H.; Mao, Y.; Mao, X.; Shi, X.; Xu, C.; Wang, C.; Zhang, S.; Zhou, D. *Advanced Functional Materials* **2018,** 28, (6), 1704855.

5. Lee, C.-H.; Lee, G.-H.; van der Zande, A. M.; Chen, W.; Li, Y.; Han, M.; Cui, X.; Arefe, G.; Nuckolls, C.; Heinz, T. F.; Guo, J.; Hone, J.; Kim, P. *Nature Nanotechnology* **2014,** 9, (9), 676-681.

6. Deng, Y.; Luo, Z.; Conrad, N. J.; Liu, H.; Gong, Y.; Najmaei, S.; Ajayan, P. M.; Lou, J.; Xu, X.; Ye, P. D. *ACS Nano* **2014,** 8, (8), 8292-8299.

7. Lee, H. S.; Ahn, J.; Shim, W.; Im, S.; Hwang, D. K. J. A. P. L. **2018,** 113, (16), 163102.

8. Cho, A.-J.; Namgung, S. D.; Kim, H.; Kwon, J.-Y. J. A. M. **2017,** 5, (7), 076101.

9. Srivastava, P. K.; Hassan, Y.; Gebredingle, Y.; Jung, J.; Kang, B.; Yoo, W. J.; Singh, B.; Lee, C. J. A. a. m.; interfaces. **2019,** 11, (8), 8266-8275.

10. Furchi, M. M.; Pospischil, A.; Libisch, F.; Burgdörfer, J.; Mueller, T. J. N. l. **2014,** 14, (8), 4785-4791.

11. Aftab, S.; Iqbal, M. W.; Afzal, A. M.; Khan, M. F.; Hussain, G.; Waheed, H. S.; Kamran, M. A. J. R. A. **2019,** 9, (18), 10017-10023.

12. Neamen, D. A., *Semiconductor physics and devices*. McGraw-Hill New York: 1997; Vol. 3.

13. Banwell, T.; Jayakumar, A. J. E. l. **2000,** 36, (4), 291-292.

14. Lee, J. H.; Gul, H. Z.; Kim, H.; Moon, B. H.; Adhikari, S.; Kim, J. H.; Choi, H.; Lee, Y. H.; Lim, S. C. J. N. l. **2017,** 17, (2), 673-678.

15. Hussain, M.; Aftab, S.; Jaffery, S. H. A.; Ali, A.; Hussain, S.; Cong, D. N.; Akhtar, R.; Seo, Y.; Eom, J.; Gautam, P.; Noh, H.; Jung, J. *Scientific Reports* **2020,** 10, (1), 9374.

16. Jaffery, S. H. A.; Kim, J.; Dastgeer, G.; Hussain, M.; Ali, A.; Hussain, S.; Eom, J.; Hong, S.; Jung, J. *Advanced Materials Interfaces* **2020,** n/a, (n/a), 2000893.

17. Ghatak, S.; Ghosh, A. *Applied Physics Letters* **2013,** 103, (12), 122103.

18. Chen, K.; Wan, X.; Xie, W.; Wen, J.; Kang, Z.; Zeng, X.; Chen, H.; Xu, J. *Advanced Materials* **2015,** 27, (41), 6431-6437.

19. Hussain, S.; Xu, K.; Ye, S.; Lei, L.; Liu, X.; Xu, R.; Xie, L.; Cheng, Z. *Frontiers of Physics* **2019,** 14, (3), 33401.

20. Zhou, N.; Wang, R.; Zhou, X.; Song, H.; Xiong, X.; Ding, Y.; Lü, J.; Gan, L.; Zhai, T. *Small* **2018,** 14, (7), 1702731.
